# Supplementary material for: Prevalence of Schistosoma mansoni infection among fishermen in Busega district, Tanzania
Source: PLoS One. 2022 Nov 28;17(11):e0276395. doi: 10.1371/journal.pone.0276395 (PMC9704623; doi:10.1371/journal.pone.0276395)

PLOS ONE

Prevalence of Schistosoma mansoni infection among fishermen in Busega district, Tanzania

--Manuscript Draft--

**Manuscript Number:**	PONE-D-22-14442
**Article Type:**	Research Article
**Full Title:**	Prevalence of Schistosoma mansoni infection among fishermen in Busega district, Tanzania
**Short Title:**	Schistosoma mansoni infection among fishermen in Busega district, Tanzania
**Corresponding Author:**	Winfrida John, MD,MSc<br>NIMR: National Institute for Medical Research-Tanzania<br>Dar es salaam, Ilala TANZANIA, UNITED REPUBLIC OF
**Keywords:**	Fishermen, Schistosoma mansoni, mass drug administration, open defecation, sanitation, intestinal schistosomiasis.
**Abstract:**	Background: Schistosoma mansoni infection is highly endemic in all regions around Lake Victoria and affects all age groups at different degrees. In most of endemic areas, less attention has been paid to determine prevalence of infection, sanitation status, and knowledge about intestinal schistosomiasis on fishermen. Therefore, the purpose of this study was to establish the prevalence of S.mansoni infection and associated factors among fishermen in Busega district.<br>Materials and Methods: A cross sectional study was conducted among fishermen in July, 2020 in five fishing villages of Busega district located along Lake Victoria. A total of 352 fishermen were interviewed by with regarding their socio-demographic characteristics, sanitation status and level of knowledge about intestinal schistosomiasis. A single stool sample from fishermen was examined for S.mansoni eggs by using Formalin-Ether Concentration technique.<br>Data were summarized into frequency and proportions then a Pearson's chi-square was used to compare proportions. In addition, logistic regression analyses were performed to determine factors associated with S.mansoni infection. The 95% Confidence interval and significance level of 0.05 was used.<br>Results: The overall prevalence of S.mansoni infection was high (65.04%) among in fishermen. Prevalence of infection varied with age of fishermen whereby fishermen aged ≤36 years had highest prevalence. Also, fishermen had low knowledge about intestinal schistosomiasis and majority of them reported to practice open defecation during fishing (81%).<br>Fishermen with the age of 37-47 years and 48-58 years had 0.411times (95% CI = 0.219 - 0.770) and 0.258 times (95% CI = 0.12 - 0.555) less likely to be infected with S.mansoni respectively. On other hand, fishermen with low knowledge about intestinal schistosomiasis had 2.747 times (95% CI: 1.044 -7.229) higher odds of being infected with S. mansoni than those with high knowledge. Equally, fishermen reported to defecate in open areas during fishing had 2.06 times (95% CI: 1.092 - 3.887) higher odds of being infected with S. mansoni than those did not reported to defecate in open areas during fishing<br>Conclusion: The prevalence of S.mansoni infection was high among fishermen in Busega district. Also, fishermen had low knowledge about intestinal schistosomiasis and reported to defecating in open area during fishing. Infection with S.mansoni was associated with age, low level of knowledge about intestinal schistosomiasis and open defecation behaviour during fishing. Therefore, mass drug administration with praziquantel, health education, sanitation behaviour change interventions were recommended for fishermen living in Busega district.
**Order of Authors:**	Winfrida John, MD,MSc
	Revocatus JL Mangar, BSc, MSc
	Billy Ngasara, MD, PhD
**Additional Information:**	
**Question**	**Response**

**Financial Disclosure**<br><br>Enter a financial disclosure statement that describes the sources of funding for the work included in this submission. Review the submission guidelines for detailed requirements. View published research articles from *PLOS ONE* for specific examples.<br><br>This statement is required for submission and **will appear in the published article** if the submission is accepted. Please make sure it is accurate.<br><br>**Unfunded studies**<br>Enter: *The author(s) received no specific funding for this work.*<br><br>**Funded studies**<br>Enter a statement with the following details:<br>• Initials of the authors who received each award<br>• Grant numbers awarded to each author<br>• The full name of each funder<br>• URL of each funder website<br>• Did the sponsors or funders play any role in the study design, data collection and analysis, decision to publish, or preparation of the manuscript?<br>• **NO** - Include this sentence at the end of your statement: *The funders had no role in study design, data collection and analysis, decision to publish, or preparation of the manuscript.*<br>• **YES** - Specify the role(s) played.<br><br>* typeset	The authors received no specific funding for this work.
**Competing Interests**<br><br>Use the instructions below to enter a competing interest statement for this submission. On behalf of all authors, disclose any competing interests that could be perceived to bias this work—acknowledging all financial support and any other relevant financial or non-financial competing interests.<br><br>This statement is required for submission	The authors have declared that no competing interests exist.

Powered by Editorial Manager® and ProduXion Manager® from Aries Systems Corporation

and **will appear in the published article** if the submission is accepted. Please make sure it is accurate and that any funding sources listed in your Funding Information later in the submission form are also declared in your Financial Disclosure statement.<br><br>View published research articles from *PLOS ONE* for specific examples.<br><br>**NO authors have competing interests**<br><br>Enter: *The authors have declared that no competing interests exist.*<br><br>**Authors with competing interests**<br><br>Enter competing interest details beginning with this statement:<br><br>*I have read the journal's policy and the authors of this manuscript have the following competing interests: [insert competing interests here]*<br><br>* typeset	
**Ethics Statement**<br><br>Enter an ethics statement for this submission. This statement is required if the study involved:<br><br>• Human participants<br>• Human specimens or tissue<br>• Vertebrate animals or cephalopods<br>• Vertebrate embryos or tissues<br>• Field research<br><br>Write "N/A" if the submission does not require an ethics statement.<br><br>General guidance is provided below. Consult the submission guidelines for detailed instructions. **Make sure that all information entered here is included in the Methods section of the manuscript.**	Ethical clearance was obtained from the Senate of Research Publication Committee of Muhimbili University of Health and Allied Sciences.<br>IRB#:MUHAS-REC-04-2020-242<br>A written informed consent was obtained from the participants before recruiting them in the study.

**Format for specific study types**<br><br>**Human Subject Research (involving human participants and/or tissue)**<br>• Give the name of the institutional review board or ethics committee that approved the study<br>• Include the approval number and/or a statement indicating approval of this research<br>• Indicate the form of consent obtained (written/oral) or the reason that consent was not obtained (e.g. the data were analyzed anonymously)<br><br>**Animal Research (involving vertebrate animals, embryos or tissues)**<br>• Provide the name of the Institutional Animal Care and Use Committee (IACUC) or other relevant ethics board that reviewed the study protocol, and indicate whether they approved this research or granted a formal waiver of ethical approval<br>• Include an approval number if one was obtained<br>• If the study involved *non-human primates*, add *additional details* about animal welfare and steps taken to ameliorate suffering<br>• If anesthesia, euthanasia, or any kind of animal sacrifice is part of the study, include briefly which substances and/or methods were applied<br><br>**Field Research**<br><br>Include the following details if this study involves the collection of plant, animal, or other materials from a natural setting:<br>• Field permit number<br>• Name of the institution or relevant body that granted permission	
**Data Availability**<br><br>Authors are required to make all data underlying the findings described fully available, without restriction, and from the time of publication. PLOS allows rare exceptions to address legal and ethical concerns. See the PLOS Data Policy and FAQ for detailed information.	Yes - all data are fully available without restriction

A Data Availability Statement describing where the data can be found is required at submission. Your answers to this question constitute the Data Availability Statement and **will be published in the article**, if accepted.<br><br>**Important:** Stating 'data available on request from the author' is not sufficient. If your data are only available upon request, select 'No' for the first question and explain your exceptional situation in the text box.<br><br>Do the authors confirm that all data underlying the findings described in their manuscript are fully available without restriction?	
**Describe where the data may be found in full sentences. If you are copying our sample text, replace any instances of XXX with the appropriate details.**<br><br>• If the data are **held or will be held in a public repository**, include URLs, accession numbers or DOIs. If this information will only be available after acceptance, indicate this by ticking the box below. For example: *All XXX files are available from the XXX database (accession number(s) XXX, XXX.).*<br>• If the data are all contained **within the manuscript and/or Supporting Information files**, enter the following: *All relevant data are within the manuscript and its Supporting Information files.*<br>• If neither of these applies but you are able to provide **details of access elsewhere**, with or without limitations, please do so. For example:<br><br>*Data cannot be shared publicly because of [XXX]. Data are available from the XXX Institutional Data Access / Ethics Committee (contact via XXX) for researchers who meet the criteria for access to confidential data.*<br><br>*The data underlying the results presented in the study are available from (include the name of the third party*	All relevant data are within the manuscript

and contact information or URL).<br>• This text is appropriate if the data are owned by a third party and authors do not have permission to share the data.<br><br>* typeset	
Additional data availability information:	

Powered by Editorial Manager® and ProduXion Manager® from Aries Systems Corporation

Manuscript Click here to access/download;Manuscript;Manuscript_Final.docx

Title: Prevalence of *Schistosoma mansoni* infection among fishermen in Busega district, Tanzania

Authors: Revocatus JL Mang'ara[1], Billy Ngasala[2], Winfrida John[2, 3]

Affiliation:

1. Department of Environmental Health Sciences, Ruaha Catholic University, Iringa, Tanzania.
2. Department of Parasitology and Medical Entomology, School of Public Health and Social Sciences, Muhimbili University of Health and Allied Sciences, Dar es Salaam, Tanzania.
3. National Institute for Medical Research, Dar es Salaam, Tanzania.

Corresponding author

Winfrida John, Email; jwinrence@gmail.com Mobile phone:+255 719826911

ABSTRACT

Background: *Schistosoma mansoni* infection is endemic in all regions around Lake Victoria and affects all age groups at different degrees. In most of endemic areas, less attention has been paid to determine prevalence of infection, sanitation status, and knowledge about intestinal schistosomiasis (KIS) on fishermen. Therefore, the purpose of this study was to establish the prevalence of *S.mansoni* infection and associated factors among fishermen in Busega district.

Materials and Methods: A cross sectional study was conducted among fishermen in July, 2020 in five fishing villages of Busega district located along Lake Victoria. A total of 352 fishermen were interviewed ~~by~~ with r~~egarding~~ their sanitation status and level of KIS. A single stool sample from fishermen was examined for S.mansoni eggs by using Formalin-Ether Concentration technique. ~~Data were analyzed by Statistical Package Social Science (SPSS) version 22 and logistic regression analyses were performed at the significance level of 0.05~~.

Results: The prevalence of *S.mansoni* infection was high (65.04%) among ~~in~~ fishermen. Prevalence of infection varied with age of fishermen whereby fishermen aged ≤36 years had highest prevalence. ~~Moreover, f~~ishermen had low ~~KIS~~ and the majority of them reported practicing open defecation during fishing (81%). These fishermen with low KIS and who reported defecating in open areas during fishing had 2.~~747~~ times (95% CI: 1.0~~44~~–7.2~~29~~) and 2.~~06~~ times (95% CI: 1.~~092~~–3.~~887~~) higher odds of being infected with *S. mansoni* than those with high ~~knowledge~~ and those who did not report defecating in open areas during fishing, respectively.

Conclusion: *S.mansoni* infection was high among fishermen in Busega district. Furthermore, fishermen had low KIS and reported to defecating in open area during fishing. Infection with *S.mansoni* was associated with age, low KIS and open defecation behaviour during fishing. Therefore, Mass drug administration with praziquantel, health educations, sanitation behaviour change interventions were needed

Keywords

Fishermen, *Schistosoma mansoni*, mass drug administration, open defecation, sanitation, and intestinal schistosomiasis.

Background

Schistosomiasis is an acute and chronic parasitic disease caused by blood flukes of the genus *Schistosoma*(1). The prevalence of schistosomiasis infection in Tanzania estimated to be 51.5%, being the second country in sub-Saharan Africa for having the highest prevalence after Nigeria (2). Poor sanitation, lack of knowledge about schistosomiasis and activities that involve high water contact like fishing are known to increased risk of schistosomiasis(2,3) S.mansoni infection is prevalent in all regions around Lake Victoria and affects all age groups at different degrees. The effort to control schistosomiasis is mainly relying on school-based Mass drug administration (MDA) which excludes adults at risk like fishermen(4,5). Currently, the magnitude of infection, sanitation status, and knowledge about intestinal schistosomiasis on fishermen remains unclear in most of endemic areas. Therefore, this study was establishing the magnitude of *S.mansoni* infection and associated factors among fishermen living in Busega district.

Materials and Methods

Study design

A cross-sectional study was carried out to determine the current magnitude of infection and associated factors among fishermen in July, 2020.

Study population

The study population was fishermen with age above 18 years and without history of taking ant-schistosomal drug within the last 3 months (incubation period of schistosomiasis).

Study area

The study was carried out in Ihale, Nyakaboja, Kalago, Nchilu and Fogofogo villages in Busega district. The district was conveniently selected because had high prevalence of 79.2% in school-aged children (6). The district is in Simiyu region located on shores of the Lake Victoria between latitude 2° 10’ and 2° 50’ South and between longitude 33° and 34° East. Fishing is a major income generating activity in the district and the district has ten (10) registered fishing boats landing sites located in a distinctive villages namely, Ijitu, Ihale, milambi, bulima, nyakaboja, mayega, kalemela, kalago, Nchilu and Fogofogo (7).

Sample size

The sample size formula for estimation of a single proportion:

$$n = \frac{Z^2 P(100 - P)}{\varepsilon^2}$$

Where, *n* = Minimum sample size, *Z* = Standard normal deviate for given confidence level (CI) = 1.96 for a 95% CI, *p* = Expected proportion =29.22% (8) and ε = Margin of error (the precision) = 5%. Calculated sample size was 318 fishermen. Assuming a 10% non-response, the resulted sample size was 353 fishermen.

Sampling

Two stage-sampling procedures were used to select villages and individual fishermen. In first stage, five ~~(5)~~ villages were selected by lottery method from a list of ten ~~(10)~~ villages. In the second stage, eligible fishermen from each of five selected villages were selected by simple random sampling technique using the list of registered names of fishermen as a sampling frame.

Number of sample fishermen from each village was determined by probability proportional to size formula: n=P*N, where, P=proportion, N=total sample size. Numbers of sample fishermen from each village were as follows: Ihale= (144/1038)353=49, Kalago= (106/1038)353=36, Nyakaboja= (92/1038)353=31, Nyamikoma 'A'= (543/1038)353= 184, Nyamikoma 'B'= (153/1038)353=52.

Data collection tools and methods

A structured interview schedule used was adapted from a study conducted in Yemen(9). The schedule was translated to Kiswahili to make it suitable to the targeted group. Data collection was carried out by the researcher and two trained research assistants accompanied by the Beach Management Unit (BMU) chairperson of the respective landing site. An interview was performed to obtain information on socio-demographic characteristics of fishermen, sanitation behavior during fishing as well as their ~~knowledge about intestinal schistosomiasis~~. Furthermore, fishermen were asked about ownership of latrines and direct observation method was used to confirm for latrine availability and type.

After interview, all participants were required to provide only one stool sample in a well labeled sterile container. The collected samples were mixed with 10% formalin (1part stool

to 3 parts preservative) to preserve the samples and to kill fecal pathogens for safety. All samples were placed in a box and transported to the NIMR Laboratory of Mwanza Centre for examination.

Stool examination

Stool samples were examined by investigator assisted by two qualified and experienced laboratory technicians. A single stool specimen per respondent was processed by ~~Formalin-Ether Concentration~~ ~~(~~FEC~~)~~ technique and double slides were prepared for each sample to examine for *S.mansoni* eggs under the microscope.

~~3.10~~ Data quality assurance

In the course of data gathering, the researcher was making a thoroughly spot-check to the research assistants to ensure correctness and completeness of the interview schedule and adherence to standard protocols of the checklist. Standard operating procedures (SOP) (10) were followed during specimen collection, transportation, processing, examination and result recording under supervision of the researcher. To verify the consistency of microscopic results, 10% of the specimens was randomly selected in each day and re-examined by researcher without prior knowledge of the results.

~~3.11~~ Data management and analysis

At the end of each day of data collection, the structured interview schedules were checked for completeness and correctness. Then, data were coded, entered into the Statistical Package for the Social Sciences (SPSS for windows, version 22.0) and cleaned for errors due to inconsistent entry. A copy of the data sheet was stored in a separate drive to save as backup. Then, the recorded data collection sheets were filled and stored.

~~Knowledge about schistosomiasis~~ was evaluated by five questions with total of 5 points. Knowledge scores were categorized as low (<2 points), average (2-3 points) and high (>3 points) (9). Data on socio-demographic, infection status, and ownership of latrine, sanitation behaviour and ~~knowledge about intestinal schistosomiasis~~ were summarized in the form of frequencies and proportions then presented in tables as well as charts.

Pearson's chi-square statistical tests were used to compare proportions between groups. Unadjusted odds ratio (UOR) was estimated by bivariate logistic regression analysis to

identify factors associated with *S.mansoni* infection to be included in the multivariate logistic regression. The biological plausible factors and all factors that were significant at significance level of 0.2 in bivariate analysis were included in the final model. Then after, adjusted odds ratio (AOR) was computed using multivariable logistic regression analysis to determine factors that were independently associated with *S.mansoni* infection. The associations were considered significant at the significance level 0.05 and 95% confidence interval (CI).

3.14 Ethical Considerations

Ethical clearance to carry out the study was obtained before conducting the study from Institutional Review Board (IRB) of MUHAS (ethics clearance no. IRB#: MUHAS-REC-04-2020-242). Permission to conduct the study in the villages was obtained from Local authorities of Busega district. Villagers were informed about the study through a village meeting and a written consent was obtained from eligible fishermen prior to recruitment into the study by using a consent form written in Kiswahili language.

RESULTS

Socio-demographic characteristics of the study participants

A total 353 eligible participants for the study, 352 fishermen from Ihale, Kalago, Nyakaboja, Nyamikoma 'A', and Nyamikoma 'B' village participated in the study while the one refused to participate. The median age was 35 years with the range of 18 to 74 years and standard deviation of 11.6 years. Majority of the participants were males (92.6%), aged ≤36 years (57.4%), married (59.4%), with primary education (84.4%) and resided in Nyamikoma 'A' village (**Table 1**).

Table 1: Socio-demographic information of fishermen participated in the study

CHARACTERISTIC	CATEGORY	N=352	%
**Age (years)**	≤36	202	57.4
	37-47	88	25
	48-58	46	13.1
	≥59	16	4.5
**Gender**	Male	326	92.6
	Female	26	7.4
**Marital status**	Not married	63	17.9
	Married	209	59.4
	Separated/divorced/Widowed	51	14.5
	Cohabiting	29	8.2
**Education level**	Completed/Not completed primary	297	84.4
	Secondary/University	55	15.6
**Village**	Ihale	49	13.9
	Kalago	36	10.2
	Nyakaboja	31	8.8
	Nyamikoma 'A'	184	52.3
	Nyamikoma 'B'	52	14.8

Prevalence of *S.mansoni* infection stratified by socio-demographic characteristics

Majority of respondents agreed to submit their stool samples 309 (87.5%) for examination. The prevalence of *S.mansoni* infection was high (65.04%) and varied significantly by age group (P <0.001), ≤36 years being the most affected age group (**Table 2**).

Table 2: Prevalence of *S.mansoni* infection in relation to demographic characteristics

VARIABLE	CATEGORY	EXAMINED (N=309)	POSITIVE (%)	P-VALUE*
**Age (years)**	≤36	177	133(75.1)	0.000
	37-47	77	41(53.2)	
	48-58	40	18(45.0)	
	≥59	15	9(60.0)	
**Gender**	Male	286	187(65.4)	0.662
	Female	23	14(60.9)	
**Marital status**	Not married	54	42(77.8)	0.184
	Married	183	113(61.7)	
	Separated/divorced/Widowed	43	28(65.1)	
	Cohabiting	29	18(62.1)	
**Education level**	Complete/Incomplete primary	259	167 (64.5)	0.633
	Secondary/University	50	34(68.0)	
**Village**	Ihale	42	27 (64.3)	0.768
	Kalago	28	20 (71.4)	
	Nyakaboja	28	16(57.1)	
	Nyamikoma 'A'	165	110(66.7)	
	Nyamikoma 'B'	46	28 (60.9)	

*P-value of Pearson Chi-square (χ^2) test

Level of ~~knowledge about intestinal schistosomiasis~~ among fishermen

The level of ~~knowledge about intestinal schistosomiasis~~ among fishermen was low (mean knowledge score of 1.97 points). The majority of the fishermen did not know or know

incorrect cause (49.4%), mode of transmission (~~76.7~~%), manifestation (90.6%) and preventive measure (~~78.7~~%) of intestinal schistosomiasis (Table ~~5~~).

Table 3: Knowledge on cause, transmission mode and clinical sign of schistosomiasis

VARIABLE	CATEGORY	N=352	%
**Cause of IS**	Worms	178	**50.6**
	Mosquito	14	4
	Snails	59	16.8
	Do not know	101	28.7
**Mode of transmission for IS**	Drinking untreated water	201	**57.1**
	Eating contaminated food	8	2.3
	Walking barefooted	7	2.0
	Swimming/bathing/fishing in Lake	82	23.3
	Do not know	54	15.3
**The main sign of IS**	Blood in urine	93	**26.4**
	Blood in stools	33	9.4
	Painful urination	85	24.1
	Stomach ache	81	23.0
	Do not know	60	17.0
**Preventive measure for IS**	Avoid fishing barefooted, swimming or bathing in the lake	75	21.3
	Avoid walking across barefooted	26	7.4
	Wash hands with water and soap	7	2.0
	Avoid drinking untreated water	147	**41.8**
	Wash fruits before eating	10	2.8
	Do not know	87	24.7
**Treatment of IS**	Traditional medicine	18	5.1

	Hospital medicine	263	**74.7**
	Do not know	71	20.2

IS= Intestinal schistosomiasis

Ownership of sanitation facilities in household and sanitation practice during fishing

Majority of fishermen had the locally designed pour flush latrines at their household and few of respondents still are using pit latrines (15.9%). Furthermore, most of the fishermen (81%) declared to defecate in open places during fishing either direct from their fishing boats or in the plastic bag then disposes it in water or in bushes along the lake (**Figure 1**).

Fig1: The place mostly used for defecation during fishing activities

Factors associated with *S.mansoni* infections among fishermen

In bivariate regression analysis, factors such as age, marital status, level of knowledge and sanitation behavior were associated with *S.mansoni* infection at significance level of 0.2.All of the above factors and a biological plausible factor (like gender)were retained in the final model.

In multivariate regression analysis, factors that were remained to associate with *S.mansoni* infection were age, low knowledge about intestinal schistosomiasis and open defecation habit. Fishermen with the age of 37-47 years and 48-58 years had 0.411times (95% CI = 0.219 - 0.770) and 0.258 times (95% CI = 0.12 - 0.555) less likely to be infected with S.mansoni respectively. On other hand, fishermen with low knowledge about intestinal schistosomiasis had 2.747 times (95% CI: 1.044 -7.229) higher odds of being infected with S. mansoni than those with high knowledge. Equally, fishermen reported to defecate in open areas during fishing had 2.06 times (95% CI: 1.092 - 3.887) higher odds of being infected with S. mansoni than those did not reported to defecate in open areas during fishing

Logistic regression analysis of infection status and risk factors

VARIABLE	CATEGORY	UOR(95% CI)	P-VALUE	AOR(95% CI)	P-VALUE
**Age (years)**	≤36	1		1	
	37-47	0.377 (0.215 - 0.661)	**0.001**	0.411 (0.219 - 0.770)	**0.006***
	48-58	0.271 (0.133 - 0.551)	**<0.001**	0.258 (0.12 - 0.555)	**0.001***
	≥59	0.496 (0.167 - 1.473)	0.207	0.489 (0.157 - 1.526)	0.218
**Gender**	Male	1		1	
	Female	0.824 (0.344 - 1.97)	0.663	1.323 (0.494 - 3.543)	0.577
**Marital status**	Not married	1		1	
	Married	0.461 (0.227 - 0.936)	**0.032**	0.814 (0.365 - 1.818)	0.616
	Separated/divorced/Widowed	0.533 (0.217 - 1.308)	0.17	0.868 (0.330 - 2.286)	0.775
	Cohabiting	0.468 (0.174 - 1.255)	0.131	0.792 (0.273 - 2.301)	0.668
**Education level**	Complete/Incomplete primary	1			
	Secondary/University	1.171 (0.613 - 2.234)	0.633		
**Village**	Ihale	1			
	Kalago	1.389 (0.494 - 3.909)	0.534		
	Nyakaboja	0.741 (0.278 -	0.548		

		1.972)			
	Nyamikoma 'A'	1.111 (0.547 - 2.258)	0.771		
	Nyamikoma 'B'	0.864 (0.364 - 2.053)	0.741		
**Knowledge level**	High	1		1	
	Average	1.468 (0.599 - 3.595)	0.401	1.460 (0.568 - 3.752)	0.431
	Low	2.829 (1.127 - 7.100)	**0.027**	2.747 (1.044-7.229)	**0.041***
**Sanitation behavior during fishing**	Not reported open defecation	1		1	
	Reported open defecation	2.177 (1.220 - 3.887)	**0.009**	2.060 (1.092 - 3.887)	**0.026***

*Significant factors with p-value < 0.05, AOR= Adjusted Odds Ratio, UOR= Unadjusted Odds Ratio

5.0 DISCUSSION

The aim of the present study was to assess the prevalence of *S.mansoni* infection and associated factors among fishermen of Busega district. We found that fishermen had high prevalence (65.04%) of S.mansoni infection in fishermen, low knowledge about and intestinal schistosomiasis and reported to practice open defecation during fishing. Furthermore, infection with S.mansoni among fishermen was associated with low knowledge about intestinal schistosomiasis and open defecation behavior during fishing.

The prevalence observed among fishermen in Busega district was higher than the prevalence of 51.8% observed among adults living in fishing villages in Mwanza region (11). The

variations in prevalence might be due to difference in study populations. Furthermore, the prevalence of *S. mansoni* infection among fishermen in Busega district was higher than ~~that~~ of 29.22% and 15.9% among fishermen at Lake Hawassa in Ethiopia (8) and at Alagoasa in Brazil (12), respectively. The difference of prevalence might be due to the variation in climate, ecology and sanitation practice of the fishermen.

The prevalence we observed in this study varied across the age groups and was higher in age ≤36 years old, similar to findings reported from other endemic areas of Tanzania (2). This may be justified by the change in water contact behaviour among young adults (13). Young people tends to change recreational swimming or playing in water body as they become adults and start carrying out adult roles (14). This result support previous studies on the need of including adult population at risk in de-worming program since may serve as a potential reservoir for re- infection of treated school children (15–17).

The level of ~~knowledge about intestinal schistosomiasis~~ among fishermen was low, and information gap exists among fishermen because most of them still hold incorrect ideas about the main sign, mode of transmission and preventive method of intestinal schistosomiasis. The confusion about sign and symptoms of intestinal schistosomiasis with that of urinary schistosomiasis was similar to previous study in Swaziland (18). Furthermore, confusion between mode of transmission and preventive method of schistosomiasis with that of soil transmitted helminths (STH) seem to be common in many endemic communities of sub-Saharan Africa (3). However, this misconception about mode of transmission ~~reported~~ to be less in some of the school children (18,19). As most of interviewed fishermen were adults, they might have not been received health education about schistosomiasis when they were at school.

Majority of fishermen reported to defecate in open places during fishing either in the bushes or direct from their fishing boats. Several studies indicated that only small group of infected people defecating in water body may create sufficient risk of exposure to all community members who will come into contact with that water later (13,17).

The present study also investigated important risk factors associated with *S.mansoni* infection among fishermen. Age, level of knowledge about intestinal schistosomiasis and sanitation habit were factors significantly associated with *S.mansoni* infection among fishermen.

Age was reported to be poor predictor of infection in the previous community study of Kenya(15). Though in the present study age was a significant predictor of infection among fishermen , whereby age > 36 years old was associated with decreased infection as compared to age ≤36 years old, similar to the previous study in Brazil(20,21). This might be due to leisure related activities in infected water sources among young fishermen as compared to older age.

Surprisingly no significant difference in infection observed between male and female fishermen. Equally, in this study gender was not a significant predictor for infection with *S.mansoni*, similar to the previous study in Kenya(15). This observation was different to the previous study reported males are more exposed and infected than females during fishing(2). The difference might be due to the fact that, fishing activity is mainly carried out by men than women in this community.

Furthermore, the present study showed that sanitation behaviour of fishermen was a significant predictor of infection, whereby fishermen who reported to practice open defecation either in bushes or in lake had increased infection compared to those did not reported open defecation practice during fishing. The findings was consistent with the previous study in Uganda (22) and in Kenya (15). Majority of fishermen reported to defecate openly during fishing and evidences of faecal materials disposed in bushes along the lake were observed during data collection. The open defecation practice in bushes might be responsible for the observed prevalence of infection among fishermen living in these areas. Therefore, decreasing community wide open defecation practice can lower the prevalence of *S.mansoni* infection in the fishing community(23).

Finding of this study also demonstrate level of knowledge about intestinal schistosomiasis was significant predictor of infection, whereby fishermen with low level of knowledge about intestinal schistosomiasis were associated with increased infection compared to those with high level of knowledge. Therefore, provision of health education to this community can

reduce prevalence of infection (23).~~The findings of the present study was similar with the study that reported from Nigeria~~(24). However, ~~it was contradicting another study conducted in Cameroon that shows a high level of knowledge about schistosomiasis was associated with the infection~~(25). This might be due health education provided to the people ~~was~~ not allow them to change their behaviour to prevent re-infection.

Limitations

The present study was subject to some limitations. Stool samples collected only on a single day to examine *S.mansoni* hence may have underestimated the prevalence of infection in the study population as parasite eggs output fluctuate day to day. Therefore, Future population studies may enhance this by collecting stool samples for at least two consecutive days. Furthermore, defecation behaviour during fishing was self-reported hence may have underestimated the actual proportion of individuals practicing open defecation in the study area.

Conclusion

In conclusion, the prevalence of *S.mansoni* infection among fishermen in Busega district was high. Prevalence of infection varied with age of fishermen whereby young fishermen (aged ≤36 years) had highest prevalence. Fishermen had low level of ~~knowledge about intestinal schistosomiasis~~ and practiced open defecation during fishing. Infection with *S.mansoni* was associated with low level of ~~knowledge about intestinal schistosomiasis~~ and open defecation behaviour during fishing.

Result of this study support the call for inclusion of fishermen in the population targeted by MDA program with praziquantel. This is important since infected fishermen may serve as potential reservoirs of *S.mansoni* infection and might be responsible for re-infection of treated school children as well as transmission of *S.mansoni* to other community groups.Also, health education should be provided in the fishing community as a supplement of MDA programs to address misconceptions about mode of transmission, symptoms and prevention of intestinal schistosomiasis.

List of abbreviation

BMU	-	Beach Management Unit
EPG	-	Eggs Per Gram.
FEC	-	Formalin-Ether Concentration
GM	-	Geometric Mean
KK	-	Kato-Katz
KIS	-	Knowledge about ~~internal~~ schistosomiasis
MDA	-	Mass Drug Administration
MUHAS	-	Muhimbili University of Health and Allied Sciences
NBS	-	National Bureau Of Statistics
NIMR	-	National Institute for Medical Research
SPSS	-	Statistical Package for Social Sciences
WHO	-	World Health Organization

Funding

Not applicable

DECLARATION

Ethics approval and consent to participate

Ethical clearance was obtained from the Senate of Research Publication Committee of Muhimbili University of Health and Allied Sciences. A permission to collect information from the district where permission to take the survey in the district was granted through the District extension officer but the information and the permission letter was sent to the respective BMU for easily reach the fishermen.

Study participants were informed of the aim of the study and its importance and written informed consent was obtained from the participants before recruiting them in the study. The information from this study was used for academic purposes only, thus each participant was treated anonymously.

Consent for publication

Not applicable as it was done under academic qualification of postgraduate education, publication is per ethical approval

Availability of data and material

The data sets used and analyzed during the current study are available and still under analysis for subsequent publications but will be available upon request from the correspond author.

Competing interests

The authors declare that they have no competing interests

Funding

This study was funded by the principal investigator himself

Authors' contributions

Revocatus JL Mang'ara designed the study, conducted data collection, did data analysis and interpretation of findings, wrote and approved the manuscript. Billy Ngasala provided technical inputs to improve designing the study, supported data analysis, read, improved and approved the final manuscript write up. Winfrida John assisted in data analysis and writing of manuscript. All authors have read and approved the manuscript to be shared for effective knowledge contribution.

Authors Information

Revocatus JL Mang'ra and Winfrida John was postgraduate student pursuing Master of Science in tropical disease control under the supervision of Professor Billy Ngasala, a Senior lecturer at Muhimbili University of Health and Allied Sciences

Acknowledgement

We would like to express our honest appreciation to all fishermen of Busega who participated in this study, together with chairperson of Ihale, Nyamikoma A and B, Nchilu and Kalago Beach management units as well as Busega district council for allowing us to carry out this study.

The special thanks goes to laboratory technicians from the National Institute for Medical Research (NIMR) Mwanza Centre, Mr. Tupevilwe Mbilinyi, Elias John and Devis Lyatuu for

technical support during laboratory examinations of stool samples and their support during data collection.

REFERENCE

1. WHO. Fact sheet on schistosomiasis. 2019. Available at: h//www.who.int/news-room/fact-sheets/detail/schistosomiasis.Accessed April 30,2022.
2. Mazigo HD, Nuwaha F, Kinung'Hi SM, Morona D, De Moira AP, Wilson S, et al. Epidemiology and control of human schistosomiasis in Tanzania. Parasites and Vectors [Internet]. 2012 Nov 28 [cited 2022 May 5];5(1):1–20. Available from: https://link.springer.com/articles/10.1186/1756-3305-5-274
3. Sacolo H, Chimbari M, Kalinda C. Knowledge, attitudes and practices on Schistosomiasis in sub-Saharan Africa: A systematic review. BMC Infect Dis [Internet]. 2018 Jan 18 [cited 2022 May 5];18(1):1–17. Available from: https://link.springer.com/articles/10.1186/s12879-017-2923-6
4. Tchuenté LT, Rollinson D, Stothard JR, Molyneux D. Moving from control to elimination of schistosomiasis in sub-Saharan Africa : time to change and adapt strategies. 2017;1–14.
5. Gouvras AN, Allan F, Kinung'Hi S, Rabone M, Emery A, Angelo T, et al. Longitudinal survey on the distribution of Biomphalaria sudanica and B. choanomophala in Mwanza region, on the shores of Lake Victoria, Tanzania: Implications for schistosomiasis transmission and control. Parasites and Vectors [Internet]. 2017 Jun 28 [cited 2022 May 5];10(1):1–14. Available from: https://parasitesandvectors.biomedcentral.com/articles/10.1186/s13071-017-2252-z
6. Siza JE, Kaatano GM, Chai J, Eom KS, Rim H, Yong T, et al. Prevalence of Schistosomes and Soil-Transmitted Helminths among Schoolchildren in Lake Victoria Basin , Tanzania. 2015;53(5):515–24.
7. BDC. Fishing Activities | Simiyu Region. Busega District council website. 2012.

8. (10) (PDF) Schistosoma mansoni infection and risk factors among fishermen at Lake Hawassa, Southern Ethiopia [Internet]. [cited 2022 May 5]. Available from: https://www.researchgate.net/publication/329430342_Schistosoma_mansoni_infection_and_risk_factors_among_fishermen_at_Lake_Hawassa_Southern_Ethiopia

9. Sady H, Al-mekhlafi HM, Atroosh WM, Al-delaimy AK, Nasr NA, Dawaki S, et al. Knowledge , attitude , and practices towards schistosomiasis among rural population in Yemen. Parasit Vectors. 2015;(August).

10. Bench aids for the diagnosis of intestinal parasites [Internet]. [cited 2022 May 5]. Available from: https://apps.who.int/iris/handle/10665/324883

11. Mazigo HD, Kepha S, Kaatano GM, Kinung'hi SM. Co-infection of Schistosoma mansoni/hepatitis C virus and their associated factors among adult individuals living in fishing villages, north-western Tanzania. BMC Infect Dis [Internet]. 2017 Oct 10 [cited 2022 May 5];17(1):1–9. Available from: https://bmcinfectdis.biomedcentral.com/articles/10.1186/s12879-017-2780-3

12. Melo AGS de, Irmão JJ de M, Jeraldo V de LS, Melo CM. Schistosomiasis mansoni in families of fishing workers of endemic area of Alagoas. Esc Anna Nery. 2018;23(1):1–10.

13. Bruun B, Aagaard-Hansen J. The social context of schistosomiasis and its control: An introduction and annotated bibliography. 2008;

14. Scott JT, Diakhaté M, Vereecken K, Fall A, Diop M, Ly A, et al. Human water contacts patterns in Schistosoma mansoni epidemic foci in northern Senegal change according to age, sex and place of residence, but are not related to intensity of infection. Trop Med Int Health [Internet]. 2003 Feb 1 [cited 2022 May 5];8(2):100–8. Available from: https://pubmed.ncbi.nlm.nih.gov/12581433/

15. Gichuki PM, Kepha S, Mulewa D, Masaku J, Kwoba C, Mbugua G, et al. Association between Schistosoma mansoni infection and access to improved water and sanitation facilities in Mwea, Kirinyaga County, Kenya. BMC Infect Dis [Internet]. 2019 Jun 7 [cited 2022 May 5];19(1):1–14. Available from: https://bmcinfectdis.biomedcentral.com/articles/10.1186/s12879-019-4105-1

16. Mazigo HD, Nuwaha F, Dunne DW, Kaatano GM, Angelo T, Kepha S, et al. Schistosoma mansoni Infection and Its Related Morbidity among Adults Living in Selected Villages of Mara Region, North-Western Tanzania: A Cross-Sectional Exploratory Study. Korean J Parasitol [Internet]. 2017 Oct 1 [cited 2022 May 5];55(5):533. Available from: /pmc/articles/PMC5678472/

17. Stothard JR, Campbell SJ, Osei-Atweneboana MY, Durant T, Stanton MC, Biritwum NK, et al. Towards interruption of schistosomiasis transmission in sub-Saharan Africa: Developing an appropriate environmental surveillance framework to guide and to support "end game" interventions. Infect Dis Poverty [Internet]. 2017 Jan 14 [cited 2022 May 5];6(1):~~1–11~~. Available from: https://idpjournal.biomedcentral.com/articles/10.1186/s40249-016-0215-9

18. Maseko TSB, Mkhonta NR, Masuku SKS, Dlamini S V., Fan CK. Schistosomiasis knowledge, attitude, practices, and associated factors among primary school children in the Siphofaneni area in the Lowveld of Swaziland. J Microbiol Immunol Infect. 2018 Feb 1;51(1):103–9.

19. Munisi DZ, Buza J, Mpolya EA, Angelo T, Kinung SM. Knowledge , attitude , and practices on intestinal schistosomiasis among primary schoolchildren in the Lake Victoria basin , Rorya District , north-western Tanzania. 2017;(September).

20. Enk MJ, Lima ACL, Barros H da S, Massara CL, Coelho PMZ, Schall VT. Factors related to transmission of and infection with Schistosoma mansoni in a village in the South-eastern Region of Brazil. Mem Inst Oswaldo Cruz [Internet]. 2010 [cited 2022 May 5];105(4):570–7. Available from: http://www.scielo.br/j/mioc/a/zs9tmQByWCkzwNQ93kwLS4y/?lang=en

21. Gazzinelli A, Velasquez-Melendez G, Crawfordb SB, LoVerde PT, Rodrigo Correa-Oliveira HK. Socioeconomic Determinants of Schistosomiasis in a Poor Rural Area in Brazil. Running short title: Socioeconomic Determinants of Schistosomiasis in Brazil. Acta Trop. 2006;99(2):260–71.

22. Exum NG, Kibira SPS, Ssenyonga R, Nobili J, Shannon AK, Ssempebwa JC, et al. The prevalence of schistosomiasis in Uganda: A nationally representative population

estimate to inform control programs and water and sanitation interventions. PLoS Negl Trop Dis [Internet]. 2019 [cited 2022 May 5];13(8):e0007617. Available from: https://journals.plos.org/plosntds/article?id=10.1371/journal.pntd.0007617

23. Hürlimann E, Silué KD, Zouzou F, Ouattara M, Schmidlin T, Yapi RB, et al. Effect of an integrated intervention package of preventive chemotherapy, community-led total sanitation and health education on the prevalence of helminth and intestinal protozoa infections in Côte d'Ivoire. Parasites and Vectors [Internet]. 2018 Feb 27 [cited 2022 May 5];11(1):1–20. Available from: https://parasitesandvectors.biomedcentral.com/articles/10.1186/s13071-018-2642-x

24. Abdulkareem BO, Habeeb KO, Kazeem A, Adam AO, Samuel UU. Urogenital Schistosomiasis among Schoolchildren and the Associated Risk Factors in Selected Rural Communities of Kwara State, Nigeria. J Trop Med. 2018;2018.

25. Sama MT, Oyono E, Ratard RC. High risk behaviours and schistosomiasis infection in Kumba, South-West Province, Cameroon. Int J Environ Res Public Health [Internet]. 2007 [cited 2022 May 5];4(2):101–5. Available from: https://pubmed.ncbi.nlm.nih.gov/17617672/

 Click here to access/download;Figure;FIGURE1.docx

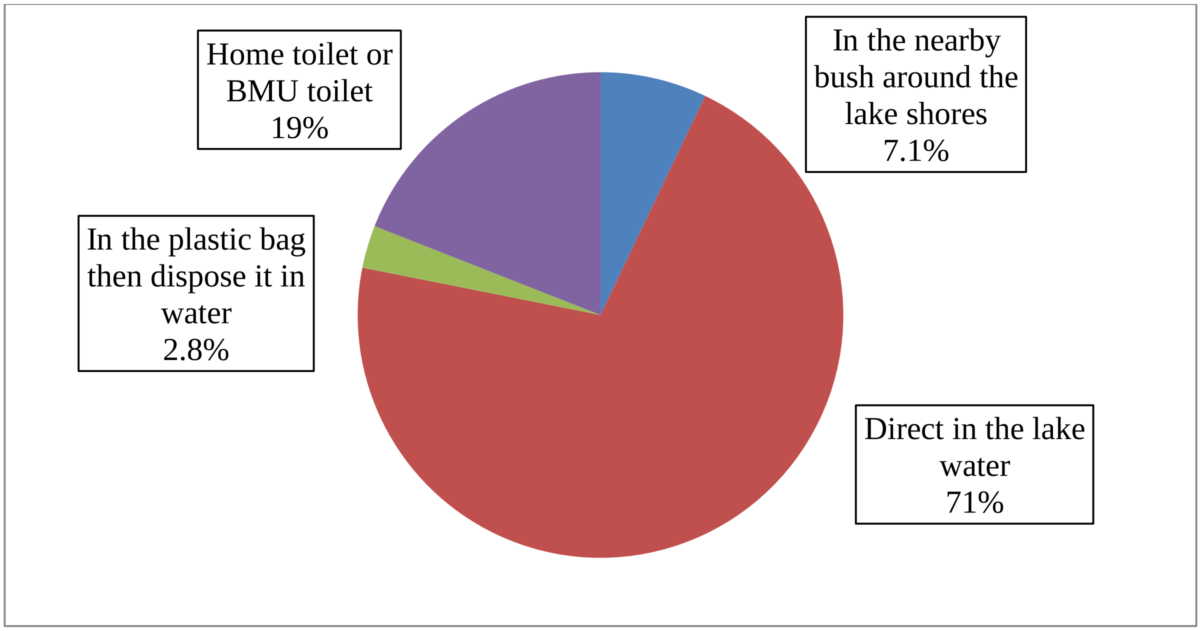

Supplement: Attachment — Submitted filename: PONE-D-22-14442_reviewer.pdf [file pone.0276395.s001.pdf]
