# Supplementary material for: Prevalence of Schistosoma mansoni infection among fishermen in Busega district, Tanzania
Source: PLoS One. 2022 Nov 28;17(11):e0276395. doi: 10.1371/journal.pone.0276395 (PMC9704623; doi:10.1371/journal.pone.0276395)
Supplement: Attachment — Submitted filename: Response to Reviewers.docx [file pone.0276395.s002.docx]

**TITLE:** **Prevalence of Schistosoma mansoni infection among fishermen in Busega district, Tanzania**

| **Comments of Academic editor and Reviewers** | **Response to Reviewers** |
| --- | --- |
| Please ensure that your manuscript meets PLOS one’s style requirements | It is written according to the PLOS one’s style requirements. |
| We suggest you thoroughly copyedit your manuscript for language usage, spelling, and grammar | A thoroughly copyedited manuscript is done accordingly. |
| Please amend either the abstract on the online submission form | The abstract has been amended as suggested. |
| Background is too short! It may be improved by providing data on global pattern of schistosomiasis in terms of number of people affected, high risk groups, and morbidities and mortality caused | The global information on schistosomiasis is included as suggested. |
| Why above 18 years? | This age group is not often targeted by MDA. |
| Did the authors collect data on frequency of fishermen' contact with water bodies? | Due to time constraints, data on the frequency of fishermen's contact with water were not collected. |
| How long were the stool samples kept before processing? | The stool samples were collected and processed the next day, so they were preserved for no more than 24 hours. |
| Please, precise the method used i.e. a stepwise backward or forward method | The enter method was used in the data regression analysis. |
| The sample size is lower than the one the authors have calculated (353). To anticipate this, they should clearly mention in the inclusion criteria that providing a stool sample was mandatory to be included into the study. Thus, in case a fisherman did not provide a stool sample, he was not included and should be replaced by another one who fulfilled the inclusion criteria. | For a variety of reasons, including a lack of time for data collection, approximately 12.5% of fishermen did not provide stool samples. This concern has been incorporated into the study's limitations. |
| How many fishermen were infected? | There were 201 infected individuals (65.04%). This information has been incorporated across the document. |
| The sum of proportions gives 100.1% instead of 100% in Table 3! Please, check it! | The 4% in Table 3 has been corrected to 3.9%. |
| Please, keep 1 digit after the decimal point!  Please, do it for all ORs! | All ORs have been corrected in the document from 3 digits after the decimal point to 1 digit. |
| Scientific names of parasites and vectors should be italicized! | The species name "*S. mansoni*" has been corrected and italicized across the document as suggested. |
| Please, go and Pubmed to complete information that is lacking in this reference | All the missing information in the reference has been corrected as suggested. |
| Why did one participant refuse to participate? | Misconceptions about the disease in general. |
